# Is passive smoking associated with acute stress response? An observational real-world study

**DOI:** 10.3389/fpubh.2026.1790527

**Published:** 2026-08-25

**Authors:** Qiongxuan Li, Rufang Gong, Qiao Wang, Hengyu Luan, Wei Lu, Xiaoguang Lu, Dongyao Li, Xiaoyong Sai

**Affiliations:** 1 Department of Statistics and Epidemiology, The Graduate School of the Chinese PLA General Hospital, Beijing, China; 2 Department of Pharmacy Intravenous Admixture Services (PIVAS), The First Medical Center of PLA General Hospital, Beijing, China; 3 Comprehensive Internal Medicine, Western Medical Branch of PLA General Hospital, Beijing, China; 4 Department of Acupuncture, Traditional Chinese Medicine Division, the Sixth Medical Center of Chinese PLA General Hospital, Beijing, China; 5 Department of Acupuncture, Traditional Chinese Medicine Division, the First Medical Center of Chinese PLA General Hospital, Beijing, China

**Keywords:** causal inference, cross-sectional survey, passive smoking, post-traumatic stress disorder, stress

## Abstract

**Background:**

This study aimed to elucidate the potential association between passive smoking and the risk of post-traumatic stress disorder (PTSD) among high-stress rescue workers, including the magnitude of such an association, and to provide a evidence-based references for the early screening, prevention, and management of PTSD risk in high-stress rescue workers.

**Methods:**

Using a cross-sectional design, the study distributed self-administered questionnaires to grassroots officers and soldiers. Data from 4,460 valid respondents were analyzed with logistic regression to quantify the association between passive smoking and positive screening under the Acute Stress Disorder (ASD) scale and PTSD Checklist for DSM-5 (PCL-5).

**Results:**

Univariate analysis showed that passive smokers had a 2.509-fold higher risk of ASD compared with non-passive smokers (OR = 2.509, 95% CI = 1.621–3.883, *p* < 0.001), and a 3.298-fold higher risk of PTSD (OR = 3.298, 95% CI = 1.459–7.451, *p* = 0.004). Multivariate analysis revealed a significant association between passive smoking and ASD risk (OR = 1.990, 95% CI = 1.959–3.144, *p* = 0.003), Passive smoking showed a strong risk association with PTSD in the partially adjusted model (model 2: OR = 3.360, 95% CI = 1.473–7.665, *p* = 0.004; model 3: OR = 3.360, 95% CI = 1.473–7.665, *p* = 0.004), but this relationship was attenuated and became non-significant in the fully adjusted model (OR = 2.513, 95% CI = 0.9–5.151, *p* = 0.085). Stratified analysis among non-smokers showed that passive smokers had a 2.487-fold (OR = 2.487, 95% CI = 1.427–4.334, *p* = 0.001) higher risk of ASD than non-passive smokers in the univariate model. Further multivariate analysis indicated that, compared with non-smokers without passive smoking exposure, the risk of ASD was significantly elevated in both the exclusive passive smoking group (OR = 1.910, 95% CI = 1.071–3.408, *p* = 0.028) and the group with both active and passive smoking (OR = 2.389, 95% CI = 1.143–4.992, *p* = 0.021).

**Conclusions:**

Significant correlations were identified between passive smoking and ASD/PTSD across the entire cohort. Specifically, among non-smokers, passive smoking exposure correlated significantly with elevated ASD risk, but its relationship with PTSD risk was indistinct. For the early screening of high-stress rescue populations, particular attention should be paid to documenting passive smoking exposure. Early intervention strategies need to prioritize reinforcing the enforcement of tobacco control policies and creating smoke-free settings, in order to mitigate the risk of ASD and PTSD linked to passive smoking exposure.

**Trial Registration:**

Clinical research registration number: ChiCTR1900023441, registered on 27 May 2019.

## 1 Background

Owing to their frequent participation in high-intensity military exercises and the limited scope of their daily social interaction, rescue personnel in grassroots forces are prone to stress, anxiety, depression, and other mental disorders, and in severe cases, even suicidal tendencies (1). High-intensity military missions are defined as military operations or deployments that subject personnel to substantial life-threatening risks, psychological trauma, and extreme stress-factors that significantly elevate their susceptibility to post-traumatic stress disorder (PTSD) (2). These missions demand not only the maintenance of peak physical performance under extreme physiological loads but also the cognitive resilience requisite for navigating complex environmental stressors.

Individuals at high risk for PTSD are characterized by heightened sensitivity and vulnerability to traumatic and stressful events, attributable to specific experiential or intrinsic factors. Typically, this high-risk cohort includes individuals with a history of combat exposure, prior trauma, distinct physiological or psychological vulnerabilities, maladaptive coping mechanisms, or comorbid psychiatric conditions (3).

Epidemiological observations reveal that grassroots rescue workers exhibit significantly higher detection rates of anxiety and depressive tendencies compared to those in logistical support roles. Furthermore, among personnel who participated in major disaster relief efforts, classic PTSD symptoms may persist for months to years (4, 5). For a subset of rescue workers, mental health symptomology is associated with acute stress and adverse life events, with onset often occurring early in their occupational trajectory (6, 7).

Accumulating evidence supports an association between PTSD and smoking behavior. Trauma-exposed individuals demonstrate substantially higher smoking rates relative to their non-traumatized counterparts, with particularly elevated prevalence among patients with PTSD (8, 9). While these findings primarily pertain to active smoking, smoking itself is frequently employed as a maladaptive coping strategy for stress mitigation (10). Notably, passive smoking exposure is disproportionately concentrated in populations at high trauma risk. Its long-term deleterious effects on mental health parallel those of active smoking, potentially contributing to an increased incidence of acute stress disorder (ASD) and other comorbid psychiatric disorders (11, 12).

Acute stress disorder (ASD) is a psychological disorder that emerges within 3 days to 1 month following exposure to a traumatic event. Its diagnostic criteria encompass intrusive symptoms, negative mood, dissociation, avoidance, and hyperarousal symptoms, accompanied by functional impairment. Notably, ASD serves as a critical predictor for the subsequent development of PTSD (13–15). Currently, research investigating the direct relationship between passive smoking and ASD remains scarce, and existing literature has not provided conclusive evidence supporting a direct association. If individuals exposed to passive smoking are also indirectly affected by nicotine, or if passive smoking environments are linked to persistent stress, they may exhibit greater psychological vulnerability following traumatic events such as high-intensity military missions. Chronic or high-level passive smoking exposure may indirectly influence the risk of developing ASD or the severity of ASD symptoms post-trauma by increasing an individual's overall psychological vulnerability and physiological stress burden (16).

Currently, smoking is highly prevalent among grassroots military rescue personnel, with a large proportion of individuals exposed to passive smoking and burdened with substantial psychological stress. Existing literature indicates that passive smoking is indirectly associated with psychological distress, anxiety, impaired cognitive function, and physiological stress responses (17, 18). Evidence supporting these indirect associations may provide clues for exploring the potential links between passive smoking and ASD, as well as PTSD (19). However, empirical research on the association between passive smoking and acute stress disorder remains inadequate, and the relationship between passive smoking and PTSD risk remains unclear due to a paucity of relevant research evidence. Therefore, this study investigates the correlation between smoking, passive smoking, and PTSD among high-stress rescue personnel, aiming to provide a reference basis for the early screening and effective intervention of PTSD in this population.

## 2 Methods

### 2.1 Participants

A cross-sectional study was conducted among rescue personnel from a unit of the Chinese People's Liberation Army (PLA) Army. A total of 5,094 grass-roots military rescue personnel were recruited From June to August 2022, with data collected on their demographic information, living conditions, and post-traumatic stress disorder (PTSD) risk status. A total of 5,094 questionnaires were distributed. After excluding invalid responses due to incomplete information, duplication, and other discrepancies, 4,460 valid questionnaires were retrieved, yielding an effective response rate of 87.55%.

The inclusion criteria were as follows: age ≥ 18 years; able to read or communicate verbally correctly without significant cognitive impairment, able to use a computer or mobile phone or complete the questionnaire with the help of others; participated in high-intensity military exercises within the past 2 weeks; and voluntarily participated and provided written informed consent following enlistment. Only those who meet all the above criteria are eligible for enrollment in this study. Participants were excluded if they met any of the following criteria: Presence of severe physical diseases such as cardiovascular, cerebrovascular, hepatic, or renal disorders; A prior diagnosis of mental illness or intellectual disability; Presence of endocrine diseases including Cushing's syndrome and Addison's disease; Pregnancy status; Presence of pituitary or adrenal tumors; Recent use of psychotropic or other medications; Failure of the participant or their legal guardian to sign the informed consent form; Agitated state precluding completion of the survey.

**Figure 1:**
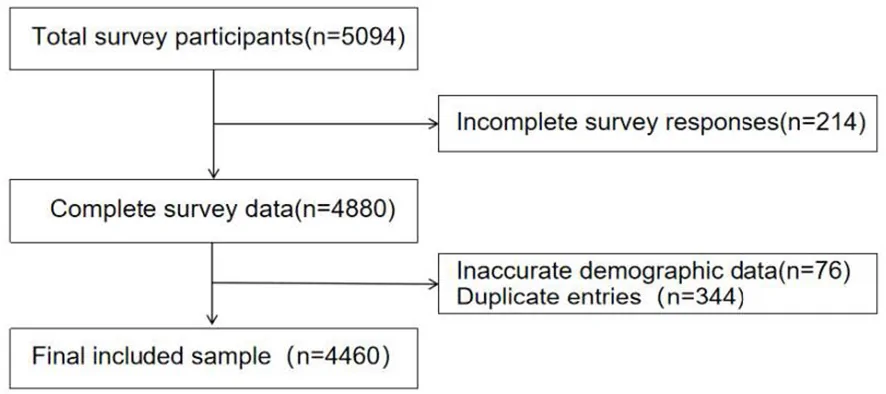
Flow diagram of participant screening.

This study received approval from the appropriate committees (Clinical research registration number: ChiCTR1900023441; Hospital ethics approval number: Institutional Review Board No. S201910801). The participant screening process is illustrated in Figure 1.

### 2.2 Data collection and assessment tools

#### 2.2.1 Data collection procedures and quality control

A cluster sampling approach was employed for this investigation. Specifically, one unit was randomly selected from over ten participating units, and all personnel within the selected unit were invited to participate. A total of 5,094 questionnaires were distributed, yielding 4,460 valid responses, resulting in an effective response rate of 87.55%.

Data were collected via an online self-administered questionnaire. The survey instrument captured information on general demographic and sociological characteristics (including gender, age, educational level, marital status, and body mass index), trauma exposure history, lifestyle factors (active smoking and passive smoking), and personal or family history of mental illness.

Stringent quality control procedures were implemented throughout the survey, as detailed below: (1) the study population consisted of rescue personnel under closed, standardized institutional management. Cluster sampling was performed based on official staff rosters, which minimized random attrition commonly seen in general population samples; (2) all survey investigators received systematic training in psychology and epidemiological fieldwork. Prior to data collection, investigators explained the study objectives and survey content to each participant, followed by written informed consent acquisition; (3) questionnaires were distributed uniformly on-site in a centralized manner with dedicated researchers providing one-on-one guidance during completion. Standardized definitions of active smoking and secondhand smoke exposure were clearly stated to unify participants' understanding of scale items and minimize measurement bias; (4) all missing or erroneous entries were corrected and supplemented in real time to exclude invalid questionnaires; (5) a random subset of 100 questionnaires was re-reviewed for consistency verification, achieving a data agreement rate of 99%; (6) two rounds of targeted follow-up reminders were delivered by dedicated researchers to participants with incomplete or blank questionnaires to maximize full population coverage; (7) raw backend survey data were manually sorted, double-entered, and independently cross-checked by specialized researchers to guarantee data authenticity and reliability; and (8) the entire survey workflow fully complied with standardized occupational epidemiological research protocols, and the high response rate observed in this study is methodologically sound and scientifically justified.

#### 2.2.2 Definition of variables

Definitions of smoking were based on the criteria established by the World Health Organization (WHO). Classification of smoking status referred to the diagnostic criteria of the Diagnostic and Statistical Manual of Mental Disorders, Fifth Edition (DSM-5), landmark studies in the field of smoking and mental health, and was consistent with the standards adopted in WHO tobacco-related research (20). A smoker was defined as an individual who had smoked continuously or cumulatively for more than 6 months in their lifetime (21); a current smoker was defined as a person who had smoked a total of 100 cigarettes and currently engaged in smoking behavior; a passive smoker was defined as a non-smoker who was exposed to sidestream smoke exhaled by smokers for at least 15 min per week on at least 1 day (22). Smoking status was categorized into four groups: (1) non-smoking with no passive smoking exposure; (2) passive smoking only; (3) active smoking only; and (4) both active smoking and passive smoking exposure.

#### 2.2.3 Assessment tools

(1) Customized scale, including basic information on rescue workers, age, sex, marital status, education level, monthly household income per capita, smoking, passive smoking, alcohol consumption, and trauma exposure.
(2) The Acute Stress Disorder Scale (ASDS) (23) is a widely used self-report instrument for assessing symptoms of ASD. Its primary purpose is to assist clinicians and researchers in rapidly identifying individuals at risk of ASD and further predicting the likelihood of PTSD. Developed based on the diagnostic criteria for ASD in the Diagnostic and Statistical Manual of Mental Disorders (DSM-IV), this scale covers the assessment of four major symptom domains: dissociation, re-experiencing, avoidance, and arousal. The scale consists of 19 self-report items, each corresponding to one of the 19 clinical symptoms of ASD specified in the DSM-IV. Participants rated the severity of each symptom using a 5-point Likert scale, where scores of 1–5 indicated “not at all,” “a little,” “moderately,” “quite a bit,” and “extremely,” respectively. The scale has been employed as a screening tool across diverse populations and traumatic events, demonstrating good reliability and validity with a Cronbach's α coefficient of 0.96.
(3) Posttraumatic Stress Disorder Checklist for DSM-5 (PCL-5) (24), which has 20 self-assessment items covering four symptom clusters: re-experiencing, avoidance, negative cognitions and moods, and hyper-arousal, and is rated on a five-point scale from 0 to 4. Higher total scores indicate more serious symptoms of PTSD. The scale has a Cronbach's alpha coefficient of 0.94, with good internal consistency.

### 2.3 Statistical analysis

We generated descriptive statistics and correlations, as well as performed independent samples *t*-test, one-way ANOVA, and chi-squared tests were performed on passive smoking of army rescue workers, using IBM SPSS Statistics for Windows, version 23.0 (authorization number: 6b4543b2xxxxf3c69a68). Quantitative data were described as *x* ± *s* or median (*M*) and interquartile range values. We used *t*-test and χ^2^ test for comparison between groups. Count data were expressed as number of cases (%). We used multiple linear and binary logistic regression to statistically analyze the influencing factors, and set *p* < 0.05 to indicate statistical significance.

## 3 Results

### 3.1 General characterization

The mean age of the respondents was 24 ± 4.1 years, the median was 23 years, and the minimum and maximum ages were 18 and 59 years, respectively. Almost all (4,396, 98.6%) of the respondents were men. Table 1 shows the descriptions of the respondents grouped by sex. Notably, excluding smokers who thought they experienced passive smoking, 11% of the respondents experienced passive smoking. Our results showed a much higher prevalence of smoking in men than in women, which is consistent with the literature (25, 26).

**Table 1:** General characteristics of study subjects (*N* = 4,460).

Variables		N (%)	Male (%)	Female (%)	*c^2^/z*	*p*
Age (years)		4,460 (100)	23 (21, 26)	25.5 (22, 28.75)	−2.966	**0.003**
Education level	University and above	2,547 (57.1)	2,495 (56.8)	52 (81.3)	19.418	**<0.001**
	High school	1,096 (24.6)	1,084 (24.7)	12 (18.8)		
	Junior college and below	817 (18.3)	817 (18.6)	0 (0)		
Marital status	Unmarried	3,661 (82.1)	3,613 (82.2)	48 (75)	2.549	0.295
	Married	779 (17.5)	763 (17.4)	16(25)		
	Other^*^	20 (0.4)	20 (0.5)	0 (0)		
Body mass index (BMI, kg/m^2^)		4,460 (100)	23 (21,24)	21 (20,23)	−4.124	**<0.001**
Monthly household income per capita (RMB)	2,999 and below	1,357 (30.4)	1,348 (30.7)	9 (14.1)	18.757	**<0.001**
	3,000–4,999	1,376 (30.9)	1,360 (30.9)	16(25)		
	5,000–7,999	928 (20.8)	912 (20.7)	16(25)		
	More than 8,000	799 (17.9)	776 (17.7)	23 (35.9)		
Smoking (No/Yes)	No	2,726 (61.1)	2,662 (60.6)	64 (100)	41.303	**<0.001**
	Yes	1,734 (38.9)	1,734 (39.4)	0 (0)		
Passive smoking (No/Yes)	No	4,012 (90)	3,956 (90)	56 (87.5)	0.433	0.51
	Yes	448 (10)	440 (10)	8 (12.5)		
Alcohol consumption (No/Yes)	No	4,131 (92.6)	4,071 (92.6)	60 (93.8)	0.011	0.915
	Yes	329 (7.4)	325 (7.4)	4 (6.3)		
Family history of mental disorders (No/Yes)	No	4,449 (99.8)	4,385 (99.7)	64 (100)		1
	Yes	11 (0.2)	11 (0.3)	0 (0)		

^*^“Other” refers to divorced, separated, widowed, etc. Bold values indicate statistically significant differences (*p* < 0.05).

### 3.2 Relation between passive smoking and ASD scale and PCL-5 scores

Among all study participants, univariate analysis showed that the risk of ASD in passive smokers was 2.509 times higher than that in non-passive smokers (OR = 2.509, 95% CI = 1.621–3.883, *p* < 0.001), and the risk of PTSD in the former was 3.298 times higher than that in the latter (OR = 3.298, 95% CI = 1.459–7.451, *p* = 0.004). Excluding the interaction effects of variables on passive smoking, the models including all influencing factors showed that passive smoking was a risk factor for developing ASD (OR = 1.990, 95% CI = 1.959–3.144, *p* = 0.003). After excluding the interaction effects of each variable on passive smoking, multivariate linear regression analysis and binary logistic regression analysis were used to statistically analyze the potential influencing factors of the study outcome by adjusting for demographic characteristics, history of alcohol consumption, family history of mental disorders, and body mass index (BMI). The results indicated that passive smoking was a risk factor for developing ASD and PTSD, with statistically significant differences in each model (ASD: model 2: OR = 2.288, 95% CI = 1.466–3.572, *p* < 0.001; model 3: OR = 2.273, 95% CI = 1.466–3.572, *p* < 0.001. PTSD: model 2: OR = 3.360, 95% CI = 1.473–7.665, *p* = 0.004; model 3: OR = 3.360, 95% CI = 1.473–7.665, *p* = 0.004). The results are shown in Table 2.

**Table 2:** Results of binary logistic regression (*N* = 4,460).

	ASD scale				PCL-5			
Variables	Model 1	Model 2	Model 3	Model 4	Model 1	Model 2	Model 3	Model 4
*B*	0.92	0.828	0.821	0.688	1.193	1.212	1.212	0.767
SE	0.223	0.227	0.227	0.233	0.416	0.421	0.421	0.445
Wald χ^2^	17.035	13.275	13.024	8.688	8.23	8.293	8.293	2.966
*p*	0^***^	0^***^	0^***^	0.003^**^	0.004^**^	0.004^**^	0.004^**^	0.085
OR	2.509	2.288	2.273	1.990	3.298	3.360	3.360	2.153
95% CI	1.621–3.883	1.466–3.572	1.455–3.550	1.959–3.144	1.459–7.451	1.473–7.665	1.473–7.665	0.9–5.151

^***^p < 0.001, ^**^p < 0.01, ^*^p < 0.05. Model 1: unadjusted; Model 2: ASD: adjusted for marital status, education level, and monthly household income. PCL-5: adjusted for sex and literacy; Model 3: ASD: adjusted for literacy, marital status, monthly household income per capita, and family history of mental disorders. PCL-5: adjusted for sex and literacy; Model 4: ASD: adjusted for sex, age, literacy, marital status, BMI, monthly household income per capita, alcohol consumption, and family history of mental disorders. PCL-5: adjusted for sex, age, education, marital status, BMI, monthly household income per capita, alcohol consumption, and family history of mental disorders.

### 3.3 Relation between passive smoking and ASD scale and PCL-5 scores in non-smokers

The number of non-smokers (*N* = 2,726) who experienced passive smoking was 301 (11%). Univariate analysis showed that the risk of ASD in passive smokers was 2.487 times higher than that in non-passive smokers (OR = 2.487, 95% CI = 1.427–4.334, *p* = 0.001), whereas the risk of PTSD in the former was 2.708 times higher than that in the latter, but there was no statistical difference (OR = 2.708,95% CI = 0.868–8.451, *p* = 0.086).

Excluding the interaction effects of variables on passive smoking, the models including all influencing factors showed that passive smoking was a risk factor for ASD (OR = 2.258, 95% CI = 1.255–4.062, *p* = 0.007). After excluding the interaction effects of each variable on passive smoking, we conducted multivariate analysis by adjusting for demographic characteristics, history of alcohol consumption, family history of mental disorders, and BMI. The results indicated that passive smoking was a risk factor for developing ASD, with statistically significant differences in each model (model 2: OR = 2.341, 95% CI = 1.325–4.136, *p* = 0.003; model 3: OR = 2.324, 95% CI = 1.311–4.119, *p* = 0.004; model 4: OR = 2.258, 95% CI = 1.255–4.062, *p* = 0.007). However, passive smoking was not significantly associated with the risk of PTSD among non-smoking individuals (model 2: OR = 2.509, 95% CI = 0.767–8.206, *p* = 0.128; model 3: OR = 2.616, 95% CI = 0.801–8.546, *p* = 0.111; model 4: OR = 2.287, 95% CI = 0.666–7.859, *p* = 0.189). The results are shown in Table 3. This finding is consistent with those of previous relevant studies and aligns with the conclusions of our research team's prior work (27, 28).

**Table 3:** Results of binary logistic regression (*N* = 2,726).

Variables	ASD				PCL-5			
	Model 1	Model 2	Model 3	Model 4	Model 1	Model 2	Model 3	Model 4
*B*	0.911	0.850	0.843	0.814	0.996	0.92	0.962	0.827
SE	0.283	0.291	0.292	0.300	0.581	0.605	0.604	0.630
waldc^2^	10.334	8.570	8.331	7.386	2.944	2.313	2.534	1.725
*p*	0.001^**^	0.003^**^	0.004^**^	0.007^**^	0.086	0.128	0.111	0.189
OR	2.487	2.341	2.324	2.258	2.708	2.509	2.616	2.287
95% CI	1.427–4.334	1.325–4.136	1.311–4.119	1.255–4.062	0.868–8.451	0.767–8.206	0.801–8.546	0.666–7.859

^***^p < 0.001, ^**^p < 0.01, ^*^p < 0.05. Model 1: unadjusted; Model 2: ASD: adjusted for education level, marital status, and monthly household income per capita. PCL-5: adjusted for literacy, marital status, and monthly household income per capita; Model 3: ASD: adjusted for literacy, BMI, monthly household income per capita, alcohol consumption, and family history of mental disorders. PCL-5: adjusted for education level, marital status, monthly household income per capita, and alcohol consumption; Model 4: ASD: adjusted for sex, age, marital status, education level, BMI, monthly household income per capita, alcohol consumption, and family history of mental disorders. PCL-5: adjusted for sex, age, marital status, education level, BMI, monthly household income per capita, alcohol consumption, and family history of mental disorders.

### 3.4 Correlation analysis of smoking with ASD scale and PCL-5 scores

Among all respondents, 127 (2.85%) screened positive for ASD on the ASD scale and 30 (0.67%) screened positive for PTSD on the PCL-5. Results of the logistic regression analysis showed that compared with non-smokers with no passive smoking, the only-passive smoking group (OR = 1.910, 95% CI = 1.071–3.408, *p* = 0.028) and both smoking and passive smoking group (OR = 2.389, 95% CI = 1.143–4.992, *p* = 0.021) were more prone to ASD risk. However, the effect of smoking status on the risk of PTSD was not statistically significant. The results are shown in Table 4.

**Table 4:** Multivariate binary logistic regression of ASD and PCL-5 (*N* = 4,460).

Variables	*N*	ASD scale				PCL-5			
		*B*	OR	95% CI	*p*	*B*	OR	95% CI	*p*
Sex	4,460 (100)	1.423	4.150	1.777–9.694	**0.001**	1.636	5.136	1.040–25.360	0.065
Age (years)	4,460 (100)	0.58	1.060	1.002–1.121	**0.042**	0.126	1.135	1.038–1.240	**0.005**
Education level					0.123				0.283
University and above	2,547 (57.1)		1.000				1.000		
High school	1,096 (24.6)	−0.516	0.597	0.362–0.986	**0.044**	−0.011	0.989	0.350–2.797	0.983
Junior college and below	817 (18.3)	−0.209	0.812	0.483–1.364	0.431	0.724	2.064	0.801–5.317	0.168
Marital status					0.426				0.620
Unmarried	3,661 (82.1)		1.000				1.000		
Married	779 (17.5)	−0.142	0.867	0.463–1.623	0.656	0.536	1.709	0.583–5.007	0.328
Other	20 (0.4)	0.867	2.379	0.495–11.439	0.279	−16.440	0.000	0.000	0.998
Monthly household income per capita (RMB)					0.829				0.178
≤ 2,999	1,357 (30.4)		1.000				1.000		
3,000–4,999	1,376 (30.9)	−0.162	0.851	0.535–1.353	0.494	−0.871	0.419	0.141–1.244	0.117
5,000–7,999	928 (20.8)	−0.171	0.843	0.502–1.414	0.517	−0.186	0.830	0.320–2.154	0.702
≥8,000	799 (17.9)	−0.238	0.788	0.450–1.379	0.404	−1.048	0.351	0.112–1.103	0.073
Alcohol consumption (No/Yes)	4,460 (100)	0.901	2.462	1.483–4.087	**0.000**	0.997	2.710	1.059–6.936	**0.038**
Family history of mental disorders (no/yes)	4,460 (100)	0.919	2.507	0.300–20.951	0.396	2.625	13.810	1.476–129.198	**0.021**
Body mass index (BMI, kg/m^2^)	4,460 (100)	−0.18	0.982	0.936–1.030	0.464	−0.058	0.944	0.839–1.063	0.341
Smoking status					0.027				0.221
No smoking + no passive smoking			1.00				1.00		
Only passive smoking		0.647	1.910	1.071–3.408	**0.028**	0.486	1.625	0.489–5.405	0.429
Only smoking		0.086	1.089	0.707–1.678	0.698	0.093	1.098	0.442–2.729	0.841
Smoking + passive smoking		0.871	2.389	1.143–4.992	**0.021**	1.254	3.503	0.988–12.422	0.052
Constants		−4.566	0.01		0.000	−7.202	0.001		0.000

“Other” includes divorce, separation, widowhood, etc. Bold values indicate statistically significant differences (*p* < 0.05).

### 3.5 Prediction model of passive smoking on ASD risk

**Figure 2:**
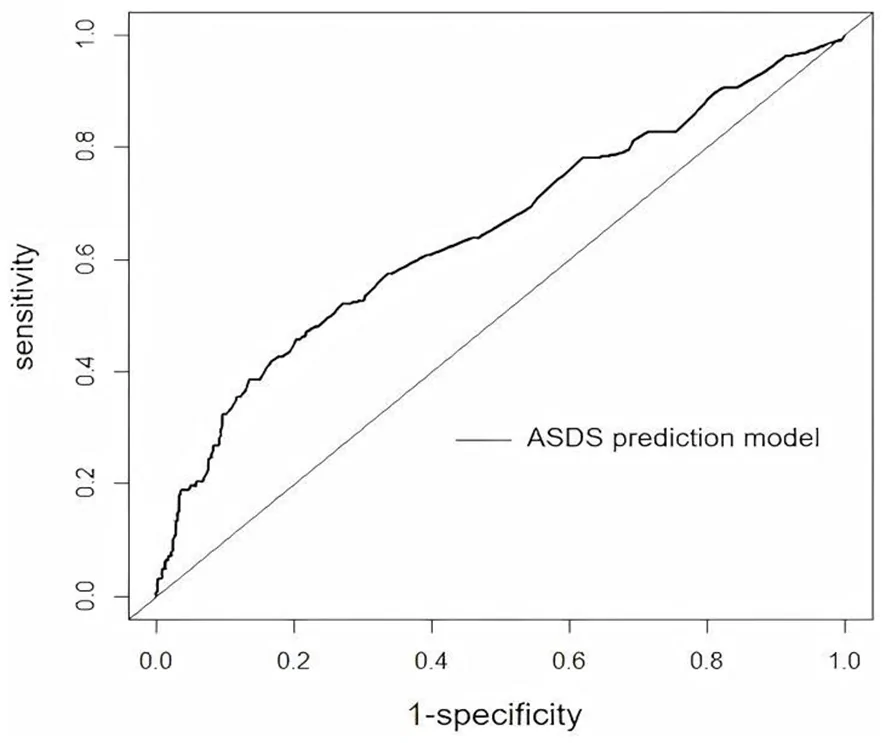
ROC curve of ASD model.

**Figure 3:**
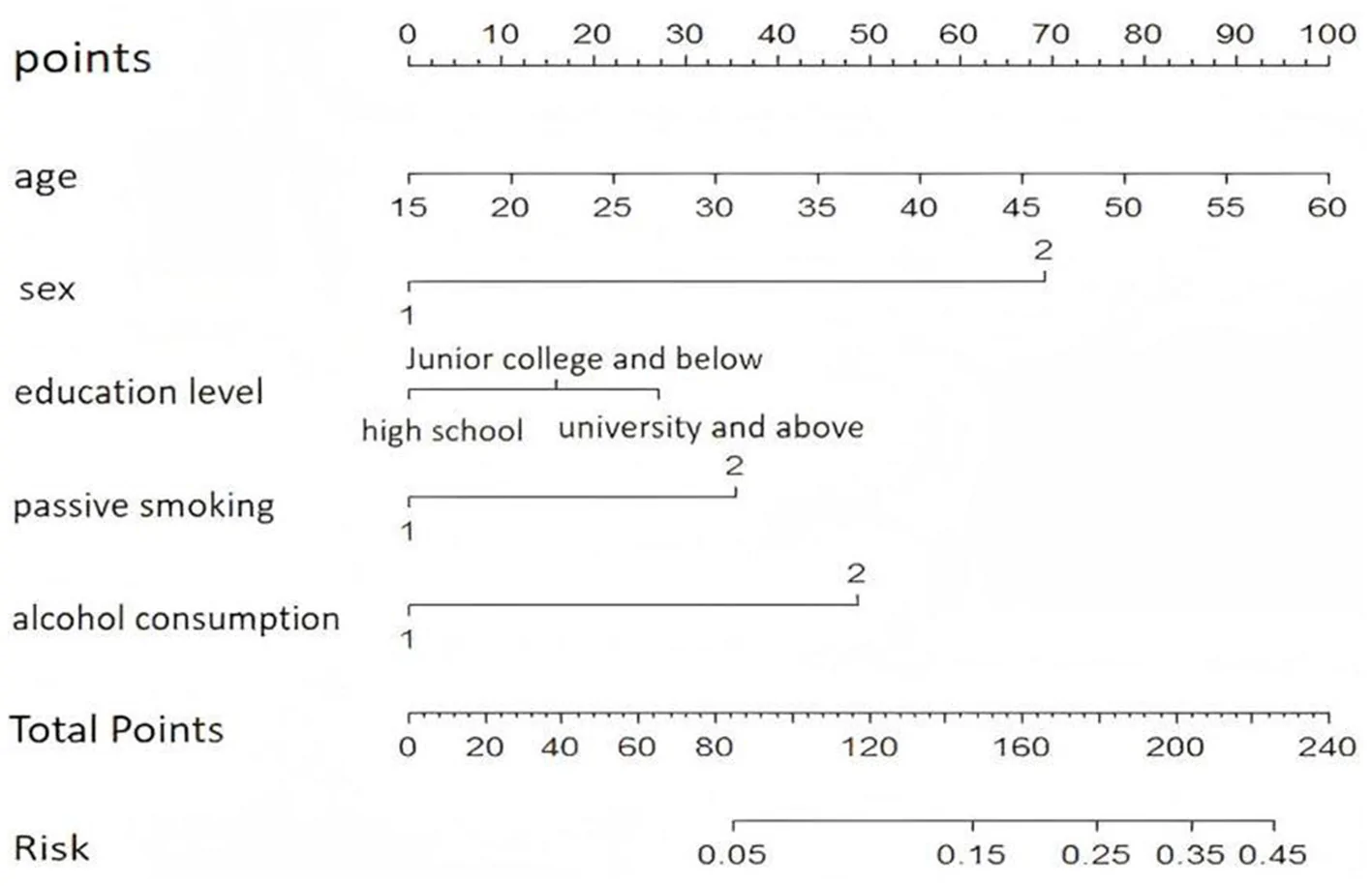
Nomogram model for predicting early risk of ASD in rescue workers.

Figure 2 shows the area under the receiver operating characteristic curve based on ASD = 0.649, 95% CI = 0.596–0.703, specificity = 0.788, and sensitivity = 0.457. Figure 3 shows the nomogram model for predicting early ASD risk, which can be used for predicting early ASD risk in high-stress rescue workers.

## 4 Discussion

High-stress rescue workers face multiple stressors. Rescuers are under great stress, and rescuers among military personnel may rely on smoking to relieve stress. However, smoking can damage their own health (29, 30) while also undermining the health of passive smokers, bringing a burden to society and families (31). Current rates of smoking and passive smoking vary by military department and position, and research on smoking and passive smoking has focused on the impact on physical diseases, such as cancer (32–34). Currently, evidence regarding the correlation between passive smoking and the risk of post-traumatic stress disorder (PTSD) development remains relatively scarce. By analyzing the prevalence of active and passive smoking among high-stress rescue personnel, this study explores the association between passive smoking exposure and PTSD risk. The objectives are to clarify the burden of tobacco smoke exposure in this population, unravel the potential correlation between passive smoking and PTSD risk, and provide a crucial reference for subsequent in-depth investigations into the strength of the association between smoking (active and passive) and PTSD risk, optimization of early PTSD screening strategies for high-stress populations, and formulation of scientific prevention and control measures.

Therefore, attention should be paid to the mental problems caused by smoking in men. A study in Greece reported that the prevalence of smoking is 38.2% among people ≥ 15 years old, and the prevalence of passive smoking at work, home, and restaurants is 52.3%, 65.7%, and 72.2% respectively (35). In Xinwu District, Wuxi City, in China, the prevalence of smoking is 21.7% and that of passive smoking is 44.02% (36). In our study, the prevalence of smoking was 38.9%, with 2,726 non-smokers, of whom 301 (11%) reported passive smoking. The discrepancy between our results and those of previous research may be related to the different populations, occupations, and stressors selected for the study.

People are aware of the effects of smoking and passive smoking on mental health (37, 38). Several studies have suggested that smoking and passive smoking are risk factors for mental disorders (39–41). Our logistic regression analysis of the total sample showed that passive smoking was significantly associated with ASD both before and after adjustment, but was associated with PTSD risk only before adjustment, suggesting that passive smoking may be a risk factor for ASD and PTSD.

In the non-smoker subgroup (*N* = 2,726), multivariate logistic regression analyses demonstrated that passive smoking exposure was strongly associated with the risk of acute stress disorder (ASD) both before and after adjustment for potential confounding factors, suggesting that passive smoking may be an independent risk factor for ASD. Based on these results, the collection of passive smoking exposure history should be prioritized in the early screening of populations at high risk of post-traumatic stress disorder (PTSD). Early intervention strategies should focus on strengthening the implementation of tobacco control measures and the establishment of smoke-free environments to reduce the risk of ASD and PTSD linked to passive smoking exposure.

Regarding smoking status, individuals who were both active smokers and exposed to passive smoking were more likely to develop ASD than those with passive smoking exposure alone, with the respective risks being 2.389-fold and 1.910-fold higher. Consequently, the risk of ASD-susceptible populations undergoing dual exposure to active smoking and passive smoking is substantially elevated. Therefore, tobacco control measures should be implemented to eliminate mental health issues induced by passive smoking among both smokers and non-smokers, thereby alleviating the burden associated with tobacco smoke exposure (42). Compared with continued smoking, smoking cessation is associated with a reduced risk of mental health problems such as depression and anxiety (43, 44). Specifically, among high-stress rescue personnel, efforts should be made to reduce both active and passive smoking rates, which will further mitigate the disease burden attributable to smoking and passive smoking exposure.

Findings derived from the multivariable regression analyses of the present study can furnish evidence-based references for the prevention and management of mental health disorders among high-stress rescue personnel: (1) Sex-stratified analysis indicated that females exhibited greater vulnerability to stress-related outcomes. Among 4,460 high-stress rescue personnel, the odds of males screening positive for Acute Stress Disorder (ASD) accounted for merely 24.10% of those in females, while the odds of males testing positive for chronic posttraumatic stress symptoms (PCL-5 positive) were only 19.47% relative to females. (2) Age-related regression results indicated that each 1-year increment in age corresponded to a 6% elevated risk of ASD and a 13.5% elevated risk of PTSD. Older participants were more susceptible to the onset of stress-related psychological symptomatology. (3) Analyses of alcohol consumption history revealed that individuals with regular alcohol use had 2.462-fold higher odds of ASD and 2.710-fold higher odds of PTSD relative to non-drinkers. This finding highlights that tobacco control interventions and alcohol-limitation health education should be integrated into standardized occupational mental health training programs. (4) Stratified analyses by family psychiatric history showed that rescue workers with a positive family history of mental illness presented 2.625 times the odds of PTSD compared to those without such familial background. We recommend implementing sustained, dynamic psychological risk surveillance for personnel with a documented family history of psychiatric disorders.

The ROC curve visualizes the discriminative capacity of the multivariable prediction model constructed by incorporating passive smoking exposure and relevant covariates for identifying ASD onset risk among high-stress rescue cohorts. The entire ROC curve lies above the diagonal reference baseline, indicating that this risk prediction model achieves acceptable overall predictive performance within the occupational study population. As a quantitative metric measuring model discrimination, the area under the curve (AUC) demonstrates that this combined model is capable of partially differentiating participants with varying ASD risk associated with secondhand smoke exposure. For occupational mental health prevention and intervention practices, this prediction model can serve as an auxiliary tool for psychological risk stratification of frontline rescue staff. Administrators may adopt the risk stratification outputs generated by the model to deliver targeted early psychological interventions and psychological resilience training for individuals classified as high-risk.

In summary, this study employed a cross-sectional design, focusing on the special occupational group of high-stress rescue personnel, to systematically analyze the association strength and potential impact characteristics between active/passive smoking exposure and the risk of post-traumatic stress disorder (PTSD). For the first time, this study clearly confirmed that passive smoking exposure is significantly associated with an increased risk of PTSD in high-stress rescue personnel. This core finding not only provides critical epidemiological evidence for the military to formulate targeted PTSD prevention and control strategies for high-stress rescue personnel but also offers a scientific basis for optimizing the occupational health support system for rescue personnel and reducing the disease burden related to psychological trauma. Additionally, given that passive smoking is a prevalent environmental risk factor, the conclusions of this study also hold certain reference value and generalizability for PTSD prevention and control in other high-stress occupational groups (e.g., emergency management personnel, firefighters, military and police officers) and the general community population.

Although the present study yielded the aforementioned valuable findings, several limitations remain to be addressed in future investigations: First, this cross-sectional study design only enables the identification of associations between passive smoking exposure and the risks of ASD and PTSD, and cannot establish definitive causal relationships or directional effects. Prospective cohort studies with long-term follow-up tracking the temporal sequence between exposure and outcomes are warranted in future work to further validate causal inferences. Second, improvements can be made regarding sample representativeness and sample size. Subsequent multi-center, large-scale epidemiological surveys are recommended, incorporating multi-dimensional exposure assessments (e.g., biomarker testing for passive smoking) and more granular adjustment for confounding factors. Such efforts will further consolidate the reliability and stability of our conclusions and furnish sufficient scientific evidence to develop targeted population-spanning prevention and control strategies for PTSD.

## Data Availability

Due to ethical constraints and institutional confidentiality requirements, the raw data cannot be publicly released. Access to relevant data is subject to institutional approval upon reasonable request.
